# Expectancy-Based Strategic Processes Are Influenced by Spatial Working Memory Load and Individual Differences in Working Memory Capacity

**DOI:** 10.3389/fpsyg.2018.01239

**Published:** 2018-07-17

**Authors:** Juan J. Ortells, Jan W. De Fockert, Nazaret Romera, Sergio Fernández

**Affiliations:** ^1^Department of Psychology, University of Almería, Almería, Spain; ^2^Department of Psychology, Goldsmiths, University of London, London, United Kingdom

**Keywords:** working memory load, Stroop priming effects, expectancy-based strategic processes, spatial working memory, individual differences in working memory capacity

## Abstract

The present research examined whether imposing a high (or low) working memory (WM) load in different types of non-verbal WM tasks could affect the implementation of expectancy-based strategic processes in a sequential verbal Stroop task. Participants had to identify a colored (green vs. red) target patch that was preceded by a prime word (GREEN or RED), which was either incongruent or congruent with the target color on 80% and 20% of the trials, respectively. Previous findings have shown that participants can strategically use this information to predict the upcoming target color, and avoid the standard Stroop interference effect. The Stroop task was combined with different types of non-verbal WM tasks. In Experiment 1, participants had to retain sets of four arrows that pointed either in the same (low WM load) or in different directions (high WM load). In Experiment 2, they had to remember the spatial locations of four dots which either formed a straight line (low load) or were randomly scattered in a square grid (high load). In addition, participants in the two experiments performed a change localization task to assess their WM capacity (WMC). The results in both experiments showed a reliable congruency by WM load interaction. When the Stroop task was performed under a high WM load, participants were unable to efficiently ignore the incongruence of the prime, as they consistently showed a standard Stroop effect, regardless of their WMC. Under a low WM load, however, a strategically dependent effect (reversed Stroop) emerged. This ability to ignore the incongruence of the prime was modulated by WMC, such that the reversed Stroop effect was mainly found in higher WMC participants. The findings that expectancy-based strategies on a verbal Stroop task are modulated by load on different types of spatial WM tasks point at a domain-general effect of WM on strategic processing. The present results also suggest that the impact of loading WM on expectancy-based strategies can be modulated by individual differences in WMC.

## Introduction

There is now a large body of evidence for a close association between working memory (WM) and selective attention (e.g., Lavie et al., 2004; Gazzaley and Nobre, 2012). Much of this evidence comes from demonstrations that WM resources are critical in achieving efficient selective behavior, which involves focusing attention on task-relevant information, while ignoring or blocking the processing of competing distractors. Studies on cognitive aging demonstrate that older adults, who usually perform worse than young adults in WM tasks (e.g., Gazzaley, 2012), also show a reduced ability to efficiently ignore and overcome the influence of irrelevant information in selective attention tasks (e.g., De Fockert, 2005; De Fockert et al., 2009; see Zanto and Gazzaley, 2014, for a review). A similar impaired performance in attention tasks (e.g., Stroop; negative priming) has frequently been observed in young adults when their cognitive resources are limited due either imposed WM load (e.g., De Fockert et al., 2001, 2010; Lavie and De Fockert, 2005; see De Fockert, 2013, for a review), or a lower WM capacity (WMC) (e.g., Kane and Engle, 2003; Kane et al., 2007; Ortells et al., 2016).

Although much less investigated, some recent studies have reported evidence that an efficient implementation of controlled facilitatory strategies like expectancy generation also relies on the availability of cognitive control resources, such as WM (e.g., Heyman et al., 2014; Hutchison et al., 2014; Ortells et al., 2017).

In a recent study, Ortells et al. (2017) used the combined WM/selective attention paradigm originally developed by De Fockert et al. (2001) in a Stroop-priming task which allows measuring of qualitatively different behavioral effects resulting from strategic vs. non-strategic processing. In this task, participants are required to identify the color (e.g., red) of a target patch which is preceded by either an incongruent (e.g., GREEN) or a congruent (RED) prime word, on 80% and 20% of the trials, respectively. As participants foreknowledged that the incongruent prime-target pairs were much more frequent than the congruent ones, and there are only two possible colors, a useful strategy would be to prepare to respond to the opposite target color to that of the prime. By implementing that strategy, participants perform much better on incongruent than on congruent trials, thus showing a reversed Stroop effect (e.g., Merikle and Joordens, 1997; Ortells et al., 2017; see also Logan et al., 1984). This Stroop task was combined with a verbal WM task of either high or low load. Participants were required to memorize sequences of digits that were presented before the prime word display, which consisted of either five repetitions of the same digit (low WM load), or five different random digits (high WM load). After performing either two, three, or four Stroop trials, participants were required to decide whether or not a single probe digit was a part of the previously memorized digit-set.

Ortells et al. (2017) found that the implementation of expectancy-based attention strategies in that version of the Stroop task critically depended on the availability of WM resources, as there was a reliable congruency by WM load interaction. Thus, when the WM task demanded a low load, participants were able to strategically process the prime to anticipate the target color, as their responses were reliably faster on incongruent than on congruent trials. This reversed Stroop effect replicates that usually observed by previous studies using this task (e.g., Merikle and Joordens, 1997; Daza et al., 2002). In clear contrast, the strategic effect was not observed when participants performed the Stroop-priming task under high WM load, as their responses were significantly slower on incongruent than on congruent trials (i.e., a standard Stroop interference effect). A similar Stroop interference effect for a highly frequent incongruent condition is usually found under task conditions that render predictive strategies difficult to implement. This is the case, for example, when a relatively short prime-target SOA interval is used in the sequential Stroop task, and/or when the prime stimulus is subliminally presented, thus impeding its conscious identification (e.g., Daza et al., 2002; Ortells et al., 2006).

The results by Ortells et al. (2017) replicate and extent those obtained by other recent studies, in showing that limiting the availability of cognitive (WM) resources with a WM task demanding a high load, can induce a less efficient strategic processing of goal-relevant information (e.g., Heyman et al., 2014; Hutchison et al., 2014).

Note, however, that in Ortells et al. (2017) study WM load was manipulated by means of a verbal task consisting of retaining sequences of digits. This memory task could encourage participants to use verbal coding strategies (e.g., rehearsal) to retain the digit set while performing the Stroop trials. Such verbal coding processes could be particularly useful during the high WM load condition, which require participants to memorize random sets of digits. If this were indeed the case, then the elimination of the strategic effect (reversed Stroop) that was reported by Ortells et al. (2017) with a high WM load could mainly reflect a greater functional overlap between the Stroop and the digit WM tasks, as both tasks would rely on verbal coding processes.

In fact, several prior studies have reported evidence that the type of concurrent WM load modulates the relative impact of cognitive load on performance in selective attention tasks (e.g., Kim et al., 2005; Park et al., 2007; see also Minamoto et al., 2015). For example, by using several variants of the Stroop task and different types of verbal and spatial WM load tasks, Kim et al. (2005) demonstrated that a higher WM load impaired selective attention processing, leading to an increased Stroop interference, when a verbal WM load was used (i.e., retaining series of letters). In clear contrast, the Stroop interference remained unaffected by a spatial WM load task (i.e., retaining the spatial locations of four randomly scattered squares) which did not overlap with either target or distractor processing in the Stroop task (see also Park et al., 2007). In contrast to load theory, which assumes that loading WM influences selective attention by disrupting general cognitive (inhibitory) control (Lavie et al., 2004), the above results rather suggest a specialized load account, according to which the impact of WM load on selective attention critically depends on whether or not load overlaps with target (or distractor) processing in the attention task.

### The Present Study

The main aim of this research is to establish whether the effects of WM load on expectancy-based strategic processes are domain-specific and limited to situations in which there is clear overlap in terms of task requirements (e.g., a digit WM task combined with a Stroop task involving color words, two tasks that likely rely on verbal coding), or whether loading WM also affects those strategic process when there is little functional overlap between the two tasks. This would suggest that the role of WM in strategic processing is relatively domain-general, for example based on shared attentional control resources.

To do so, in two experiments we used different types of spatial memory tasks to load WM while observers performed a strategic Stroop task. Our predictions were that, if WM plays a domain-general role in expectancy-based strategic processing, then loading non-verbal spatial WM should modulate verbal Stroop effects. Conversely, if the role of WM in expectancy-based strategic processing is more domain-specific, then the lack of functional overlap between spatial WM task and the Stroop task should mean that loading WM in the present study will modulate the strategic Stroop effect to a lesser degree than we found when using a verbal WM task (Ortells et al., 2017). Indeed, previous work investigating effects of verbal vs. non-verbal WM load on visual detection found opposite effects of load on detection of a task-unrelated visual stimulus, with an improved detection under high verbal WM load, and a reduced detection under high visual WM load (Konstantinou and Lavie, 2013).

It is also interesting to note that whereas a reliable reversed Stroop in the Stroop-priming task was observed by Ortells et al. (2017) when the concurrent verbal WM task demanded a low load, this was not the case for all participants in their study. Further data inspection revealed that more than a third of their participants (nine out of 26 participants in the study) showed a conventional Stroop interference effect not only with a high WM load, but also with a low WM load. It appears that these participants were unable to strategically anticipate the target color (i.e., the opposite to that of the prime word) even when the WM task demanded a low load.

This pattern of inter-individual differences resembles that observed by Froufe et al. (2009) between young and elderly people. In this study, two groups of older adults (one with Alzheimer’s dementia – AD), and one group of younger adults carried out a sequential Stroop task very similar to that of Ortells et al. (2017) (but under single-task conditions), as the proportion of incongruent prime-target pairs was much higher (84%) than that of congruent pairs (16%), and participants were informed of these proportions at the beginning of the experiment. Froufe et al. (2009) found that the younger adults responded reliably faster to the incongruent than to the congruent targets (reversed Stroop), which confirms that they were able to efficiently implement expectancy-based strategic actions in this task. In clear contrast, a non-significant reversed Stroop was found in elderly people without AD, whereas the older adults with AD responded significantly slower to incongruent than to congruent targets (standard Stroop interference). This later finding suggests that, in addition to any decline in strategic processing associated with normal aging, AD is associated with a further reduction in capacity to implement expectancy-based strategies.

Based on these results, one could speculate that healthy young adults showing Stroop interference, instead of reversed Stroop, under the low load condition in Ortells et al. (2017) study, could have had lower WMC than the remaining participants who showed a reversed (strategic) Stroop with a low WM load. However, WMC of participants was not assessed by Ortells et al. (2017). Whereas a few previous studies have examined the combined effect on performance of limiting WM by both imposed WM load and individual differences in WMC (e.g., Rosen and Engle, 1997; Kane and Engle, 2003; Ahmed and De Fockert, 2012), to our knowledge, the interactive impact of these two factors on strategic processing has not been investigated previously. Consequently, a second aim of the present research was to explore whether individual differences in WMC could modulate the impact of loading WM on expectancy-based strategic processes.

To this end, participants in our experiments also performed a change localization task (e.g., Johnson et al., 2013). On each trial a sample array containing four colored shapes was briefly presented (e.g., 100 ms), and followed after a short delay (e.g., 900 ms) by a test array, which was similar to the previous sample display except that one of the four items had changed colors, and participants had to select the location of the change. This is a very simple task in which there is no task switching or time pressure, and guessing effects are minimized by the fact that chance level is 25% instead of 50% (Johnson et al., 2013). But importantly, like it is the case with complex span tasks frequently used to asses WMC (e.g., Operation Span Task), performance in the change detection/localization tasks has been shown to have strong relationships with broader measures of higher cognitive abilities, including fluid intelligence, and attention control capacities, in both healthy adults and several clinical (e.g., people with schizophrenia) populations (e.g., Cowan et al., 2005; Fukuda et al., 2010; Johnson et al., 2013; Shipstead et al., 2015).

## Experiment 1

We used in this experiment the same Stroop-priming task as the one used by Ortells et al. (2017), but this task was now combined with a non-verbal (spatial) WM task of either low or high load. The memory set preceding the prime word consisted of four arrows, the orientation of which had to be retained by participants (see Chao, 2011, Experiment 7, for a similar spatial WM task). The four arrows could either all point in the same direction (low WM load condition) or in different random directions (high WM load condition). After performing a variable number (two, three, or four) of Stroop-priming trials, a single probe arrow was displayed and observers were required to decide whether or not that arrow had been presented in the previously memorized arrow-set. To the extent that the effects of loading spatial WM on expectancy-based strategies are mainly domain-general (e.g., based on shared attentional resources) rather than domain-specific, we again expected to find a Stroop interference effect when the spatial WM task would involve a high load. By contrast, a reversed strategic Stroop effect should be observed when the load of the spatial WM task was low.

On the other hand, to the extent that strategic planning for a likely target under dual-task conditions requires that cognitive control resources are maximally available, that is, under low WM load and in high WMC individuals, we expect to obtain a reliable three-way interaction between prime-target congruency, WM load and WMC. In line with previous findings by Ortells et al. (2017), we predict that under high WM load all participants, regardless of their WMC, will be unable to efficiently ignore the incongruence of the prime and therefore show a standard Stroop effect. When the load of the concurrent WM task is low however, the ability to ignore the incongruence of the prime could be modulated by WMC, such that a reversed Stroop effect should be found in participants with a higher WMC.

### Materials and Methods

#### Participants

Forty-four right-handed undergraduate students (28 women; age range = 19–30 years, *M* = 20.73, *SD* = 2.54) from the University of Almería received course credits for their participation in the experiment, with all them having normal or corrected-to-normal vision. The sample size was greater than that used by previous studies using this strategic Stroop-priming task (e.g., Froufe et al., 2009; *n* = 27; Ortells et al., 2017; *n* = 26), and very similar to that used by other studies that had addressed the combined effect on performance of both WM load and individual differences in WMC (e.g., Ahmed and De Fockert, 2012; *n* = 43). The experiments of the present research were conducted in compliance with the Helsinki Declaration, and with the ethical protocols and recommendations of the “Code of Good Practices in Research,” “Commission on Bioethics in Research from the University of Almería.” All participants in this and the remaining experiment signed informed consents before their inclusion, with the protocol being approved by the “Bioethics Committee in Human Research” from the University of Almería.

#### Stimuli and Apparatus

**FIGURE 1:**
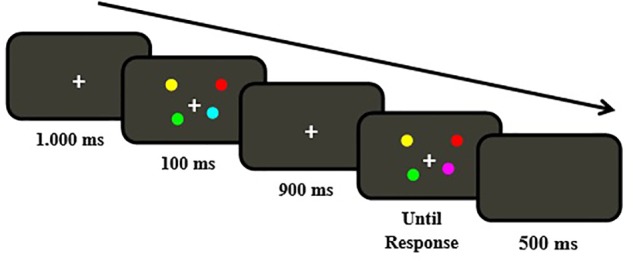
Sequence of events of a trial in the change localization task.

The stimuli were displayed on a 17-in. CRT monitor controlled by a computer running E-prime 2.0 software (Psychology Software Tools). Viewing distance was approximately 60 cm. In the change localization task, participants were presented with visual arrays containing four colored circles displayed against a gray background (60, 60, 50), with each circle subtending a diameter of about 0.96° (**Figure 1**). The four colors were randomly selected from a set of nine different colors with the following red, green, and blue values: black (0, 0, 0), blue (0, 0, 255), cyan (0, 255, 255), green (0, 255, 0), magenta (255, 0, 255), orange (255, 113, 0), red (255, 0, 0), white (255, 255, 255), and yellow (255, 255, 0). The four colored circles presented on each trial were randomly displayed in each of the four quadrants of the screen, with the distance between fixation and the nearest and farthest circles subtending about 3.36° and 4.8°, respectively.

**FIGURE 2:**
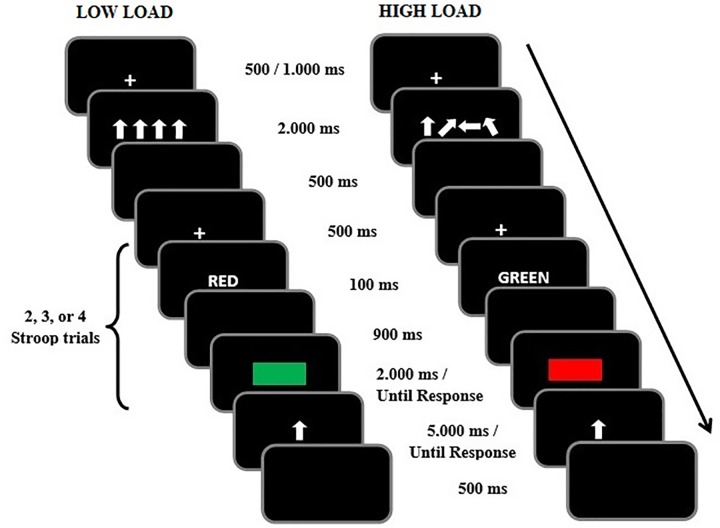
Examples of incongruent trials in the Stroop task under low **(Left)** and high **(Right)** working memory load in Experiment 1.

The experimental trials of the WM/Stroop-priming task consisted of a WM (arrow direction recall) and an attention (Stroop-priming) component (see **Figure 2** below for sample trial sequences). For the WM component, sets of four arrows pointing in eight possible different directions (up, down, left, right, up-left, up-right, down-left, down-right) were centrally displayed in white in a horizontal line, with each arrow subtending a visual angle of about 0.76° wide and about 0.96° high. In the low WM load condition, the four arrows pointed in the same direction. In the high WM load condition, the four arrows pointed in four different directions, which were generated randomly from the eight possible directions. The memory probe consisted of a centrally presented single white arrow. For the Stroop-priming component, the prime stimuli consisted of the color words ‘ROJO’ (RED) or ‘VERDE’ (GREEN) displayed in white color in Courier new font size 22 (each character at about 0.35° wide and 0.52° high). The target consisted of a rectangle displayed in either red (255, 0, 0) or green (0, 255, 0) color at fixation, and subtending about 7.39° horizontally and 2.6° vertically. All stimuli presented in the WM/Stroop-priming task were displayed against a black background.

#### Design and Procedure

Participants performed a single experimental session lasting about 40–45 min. Each participant first completed a version of the change localization task (e.g., Johnson et al., 2013) to measure their WMC. Each trial started with a central fixation point (+) that remained on the screen until the end of the trial. After 1,000 ms, a sample array displaying four colored circles (each circle colored in a different color) was presented for 100 ms. After a 900 ms blank screen, a test array appeared, which was similar to the previous sample array except that one of the four circles had changed color, and participants had to indicate the location of the change using the computer mouse (**Figure 1**). Participants performed 12 practice trials and two experimental blocks of 32 trials per block, with a break interval between the two experimental blocks. A variant of the Pashler/Cowan K equation (e.g., Cowan et al., 2005) was used to assess participants’ WMC. As each stimulus array contains four circles and each test array always contains a circle that changed color, the proportion of correct responses from each participant was multiplied by four to calculate their WMC (*K* score).

After completing the change localization task, each participant performed the combined WM/Stroop-priming task. The timing of the specific stimulus events on each trial was as follows: (1) Fixation display (+) presented for a variable duration (500–1,000 ms); (2) Memory set presented for 2,000 ms, which contained four arrows pointing in either the same (low WM load) or different directions (high WM load); (3) Blank screen presented for 500 ms; (4) Stroop-priming trials (see below for details); (5) Memory probe display (a single arrow) presented for 5,000 ms or until response. Participants had to decide whether or not the arrow probe had been present in the previously memorized arrow-set by pressing the ‘1’ or ‘2’ keys with the middle and index fingers of their left hand, respectively (key mappings counterbalanced across participants). The probe arrow was either present or absent in the memory set on the same number of trials, and when it was present, it could occur with the same probability in any of the four positions. Following the participant’s response to the arrow probe a new trial began after an inter-trial interval (blank screen) of 500 ms.

On each WM trial and following the memory set, participant performed a variable number (two, three, or four) of Stroop trials, with the timing of the specific stimulus events on each Stroop trial being as follows: (1) Blank screen presented for 500 ms; (2) Prime word [‘ROJO’ (RED) or ‘VERDE’ (GREEN)] displayed for 100 ms (in white letters); (3) Blank screen presented for 900 ms; (4) Target stimulus (a red or green central rectangle) which remained on the screen until response. The participants responded to the rectangle color by pressing the ‘b’ and the ‘n’ keys with the index and middle fingers of their right hand. The two keys were labeled RED and GREEN with red and green stickers (key-label mappings counterbalanced across participants). The response to the target was followed by either the next Stroop trial, or the memory probe display. The prime and target stimuli referred to either the same color (congruent) or different colors (incongruent) on 20% and 80% of the trials, respectively. At the beginning of the experiment, participants received information about that differential proportion of congruent and incongruent pairs, and were actively encouraged to strategically use that information to optimize their performance in the Stroop task.

The combined WM/Stroop-priming task included 36 practice trials (18 for low and 18 for high WM load) followed by 180 experimental trials divided in two blocks, with 90 trials for each WM load condition (with the order of the two load blocks being counterbalanced across participants). There were 30 WM trials for each load block, with a same number of WM trials (10) containing either two, three, or four Stroop-priming trials (each participant received a different random order of the 30 WM trials). The 90 Stroop trials of each WM load block included 72 incongruent (80%), and 18 congruent (20%) trials. Once a WM load block was initiated, it ran to completion.

### Results and Discussion

Participants’ responses to the memory probe showed the effectivity of our WM load manipulation. Mean correct RTs to the arrow probe were significantly slower in the high WM load (*M* = 2007 ms; *SD* = 522) compared to the low WM load block [*M* = 1688 ms; *SD* = 457; *t*(43) = 4.68, *p* < 0.001; *d* = 0.65]. Mean accuracy was also reliably lower in the high (*M* = 0.70; *SD* = 0.11) than in the low WM load condition [*M* = 0.93; *SD* = 0.062; *t*(43) = 15.12, *p* < 0.001; *d* = 2.41]. The results of further ANCOVA analyses in which *K* scores in the change localization task were treated as a continuous covariate, showed no reliable interaction between WM load and WMC either in reaction times [*F*(1,42) = 1.3, *p >* 0.26] or in response accuracy (*F* < 1), thus suggesting that memory task performance was not modulated by individual differences in WMC (see Ahmed and De Fockert, 2012; Experiment 1, for a similar result).

For the analysis of responses in the Stroop task, were excluded trials with target responses that were incorrect (1.78%) or faster than 200 ms (0.47%). In addition, we included in this analysis only those trials on which the response to the arrow memory probe was correct. Mean RTs and error rates were entered into two 2 × 2 Analyses of Variance (ANOVAs), with WM load (low and high) and prime-target congruency (congruent and incongruent) and as within-participants factors^1^. Mean correct RTs and error rates as a function of congruency and WM load conditions are depicted in **Table 1**.

**Table 1:** Mean (SD) correct reaction times (ms) and error percentages (in %) for congruent and incongruent trials in the Stroop task, under low and high WM load in Experiment 1.

	Prime-target congruency		
	Congruent	Incongruent	Stroop-priming
Working memory load		
Low load	546 (111.2) 1.09 (2.9)	550 (100.8) 1.02 (2.2)	−4
High load	559 (114.4) 1.11 (3.2)	603 (106.9) 1.18 (3.3)	−44

The ANOVA on error rates revealed no reliable effects (all *F*s < 1). The RT ANOVA showed a significant effect of WM load [*F*(1,43) = 5.57, *p* = 0.023, η^2^ = 0.11], such that responses were slower in the high load (*M* = 581 ms) than in the low WM load condition (*M* = 548 ms). The main effect of congruency reached also significance [*F*(1,43) = 6.88, *p* = 0.012, η^2^ = 0.14], with slower responses on incongruent (*M* = 576 ms) than on congruent (*M* = 552 ms) trials (i.e., a standard Stroop interference effect). In addition, the two factors reliably interacted [*F*(1,43) = 6.02, *p* = 0.018, η^2^ = 0.12], such that different Stroop effects emerged for high and low WM load conditions. Imposing a high load on the WM task induced reliably slower responses (by 44 ms) on incongruent than on congruent trials in the Stroop-priming task [*t*(43) = 3.28, *p* = 0.002, *d* = 0.496]. Whereas this latter finding replicates that reported by Ortells et al. (2017) with a verbal WM task, no reliable reversed Stroop effect was found when our WM task demanded a low load (*t* < 1; **Table 1**).

**FIGURE 3:**
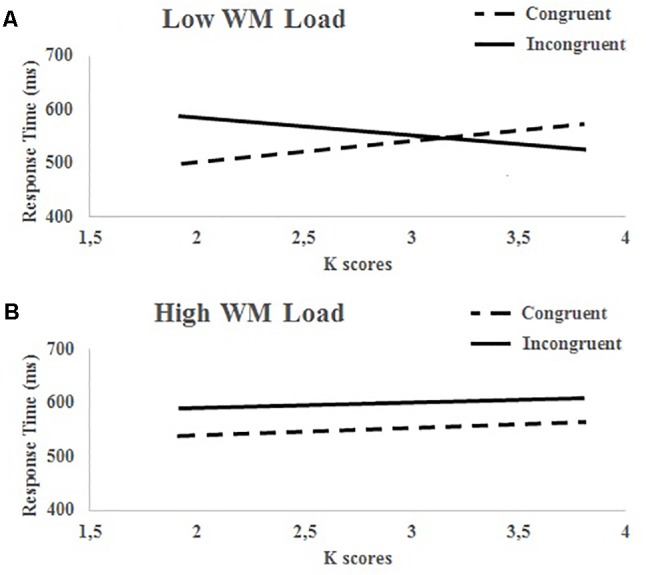
Participants’ response times (ms) for congruent and incongruent conditions in the Stroop task as a function of WMC (*k*) scores under low **(A)** and high **(B)** WM load in Experiment 1.

In order to know whether the strategic use of congruency proportion in the Stroop-priming task was modulated by individual differences in WMC, we conducted a further ANCOVA treating WM load and congruency as within-participants factors, and WMC (*K* scores) as a continuous covariate variable (for similar analyses, see Hutchison, 2007; Richmond et al., 2015). The results showed again a main effect of prime-target congruency [*F*(1,42) = 5.84, *p* = 0.02, η^2^ = 0.12], which was qualified by a reliable congruency × WMC interaction [*F*(1,42) = 4.13, *p* = 0.049, η^2^ = 0.09], and of more interest, by a WM load × Congruency × WMC three-way interaction [*F*(1,42) = 4.27, *p* = 0.045, η^2^ = 0.092]. To decompose this latter interaction, we analyzed the single congruency × WMC interaction separately for high and low WM load conditions (**Figure 3**).

**FIGURE 4:**
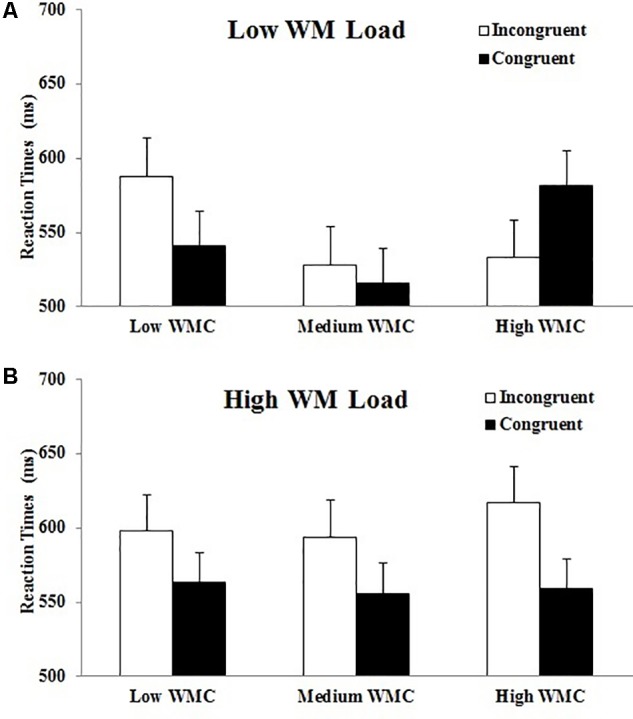
Mean reaction times (and standard error of the mean) for congruent and incongruent prime-target pairs as a function of WM load (**A**: low load; **B**: high load) and WMC group (low-, medium-, and high-WMC) in Experiment 1.

As shown in **Figure 3**, under a high WM load no reliable congruency × WMC interaction was ever found (*F* *<* 1), with participants consistently showing an interference Stroop effect irrespective of their WMC (see also **Figure 4**^2^). Under a low WM load, however, there was a reliable crossover interaction between congruency and WMC [*F*(1,42) = 12.24, *p <* 0.001, η^2^ = 0.23], which shows that only participants with higher WMC were capable of an efficient strategic use of congruency proportions, giving rise to a reversed Stroop-priming effect. In clear contrast, participants with lower WMC showed an opposite Stroop interference effect, even though the concurrent WM task imposed a low load. Thus, the probability to find an expectancy-based priming effect (i.e., reversed Stroop) is positively correlated with WMC under a low WM load (*r* = 0.46, *p* = 0.002) but not under high WM load (*r* = 0.002, *p* >0.88).

## Experiment 2

In Experiment 1, we interleaved the strategic Stroop-priming task used by Ortells et al. (2017) with a WM load task which required participants to memorize the spatial directions of four arrows pointing either in a same direction (low load) or in four different random directions (high load). Although this non-verbal WM task was similar to that used in other previous studies (e.g., Chao, 2011; Experiment 7), it could however be questioned whether this particular task was truly spatial. Note on this respect that in both high and low load conditions, the four arrows always appeared in fixed spatial locations and they were ordered from left to right similarly to verbal information. Given those presentation conditions, one could argue that participants in our experiment might still be using some kind of verbal coding strategy to memorize the arrow sets. For example, they could use verbal rehearsal of lists of directions words like “up, up, up, up,” and “up, right-up, left, left-up,” to retain in verbal WM the low and high WM sets presented in **Figure 2**, respectively^3^. If that was really the case, then it would be difficult to establish whether the impact of WM load on expectancy-based strategic processes that was found in our experiment, was truly reflecting a domain-general, rather than a more domain-specific effect.

Based on these lines of argument, in the present experiment we used a different WM loading task that involved stimuli that are more unequivocally spatial and non-verbal than those used in Experiment 1. Accordingly, our Stroop task was now combined with a WM task that required observer to memorize the spatial locations of four dots presented in a 4 × 4 square grid. In a low load condition, the four dots always form a symmetrical pattern (i.e., a straight line), whereas in a high load condition, they are randomly scattered in the square grid. After running 2, 3, or 4 Stroop-priming trials, a single memory probe dot is presented in the square grid, and participants had to decide whether it is occupying or not any of the four spatial locations previously occupied by the remembered dots. This kind of WM loading task has been used by several prior studies to investigate whether attentional processes can be affected by load manipulations in a concurrent spatial WM task (e.g., Smith and Jonides, 1998; Kim et al., 2005; Heyman et al., 2014; see also Thomas, 2013).

### Materials and Methods

#### Participants

Forty right-handed undergraduate students (12 men; age range = 19–33 years, *M* = 21.42, *SD* = 3.21) from the University of Almería received course credits for their participation in the experiment, with all them having normal or corrected-to-normal vision.

#### Stimuli and Apparatus

These were similar to those used in Experiment 1, with the only difference being the WM component of the combined WM/Stroop-priming task. For the WM component, a 4 × 4 square grid (about 10.56° wide and high) containing four black filled dots (1.44° diameter) was centrally displayed. The four dots either formed a simple symmetrical pattern (i.e., a straight line; low WM load condition), or they were randomly scattered in different spatial locations in the square grid (high WM load condition), with the restriction that the dots had no adjacent neighbors in either vertical or horizontal directions. The memory probe consisted of a square grid containing a single black filled dot (1.44° diameter).

#### Design and Procedure

**FIGURE 5:**
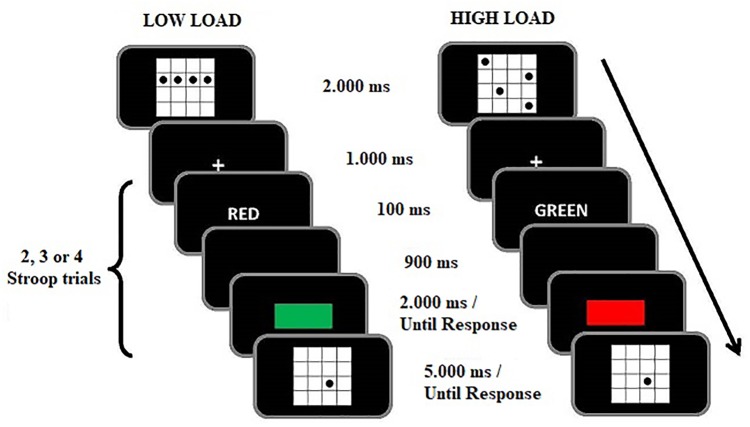
Examples of trials under low **(Left)** and high **(Right)** load in the spatial working memory task in Experiment 2.

These were the same as those used in Experiment 1, with the difference that the WM loading task now consisted of memorizing the spatial locations of four dots that were simultaneously displayed in a 4 × 4 square grid for 2,000 ms. In the low WM load condition, the four dots formed a straight line (**Figure 5**), whereas in the high WM load trials, the dots were randomly displayed in the square grid (Kim et al., 2005; Heyman et al., 2014, for similar spatial WM load tasks). After performing two, three, or four Stroop trials, a single dot was present for 5,000 ms or until response in the square grid. Participants had to press the ‘1’ or ‘2’ keys to decide whether the probe dot either appeared in one of the locations occupied by the memorized dots or it was presented in a different (unoccupied) location to those of the memorized dots (key mappings counterbalanced across participants). Following the participants’ responses to the dot probe a blank screen was presented for 500 ms (inter-trial interval). The dot probe was equally likely to appear in either the same location or a different location to those of the memorized dots. As in Experiment 1, participants knew that the incongruent trials were much more frequent (80%) than the congruent trials (20%) in the Stroop task, and were encouraged to strategically use the prime word to anticipate the target color. The combined spatial WM/Stroop-priming task again included 36 practice (18 for each WM load condition) and 180 experimental trials divided in two blocks: 90 trials for the high WM load and 90 for the low WM load block (block order counterbalanced between participants). Participants performed 30 WM trials of each load block, and each WM trial included two, three or four Stroop trials (10 WM trials each).

### Results and Discussion

Participants’ responses to the memory probe demonstrated again the effectivity of our manipulation to load spatial WM. Mean correct response times to the dot probe were significantly slower in the high WM load condition (*M* = 1809 ms; *SD* = 525) compared to the low WM load condition [(*M* = 1647 ms; *SD* = 302; *t*(39) = 2.67, *p* < 0.011; *d* = 0.42]. Mean accuracy was also reliably lower for high (*M* = 0.79; *SD* = 0.10) than for low WM load trials [*M* = 0.93; *SD* = 0.06; *t*(39) = 9.02, *p* < 0.001; *d* = 1.47]. The results of further ANCOVAs treating participants’ *K* scores in the change localization task as a continuous covariate, showed that WMC did not interact with WM load in response times to the memory probe [*F*(1,38) = 1.59, *p >* 0.215], as found in Experiment 1. Yet, the WM load by WMC interaction reached statistical significance in probe accuracy rates [*F*(1,38) = 7.63, *p* = 0.009, η^2^ = 0.17]. The analysis of this interaction showed that a greater WMC was associated with a decreased difference in accuracy rates between low and high WM conditions, as revealed by a reliable negative correlation between both variables (*r* = -0.40, *p* = 0.012). A similar interaction between WM load and WMC in probe response accuracy has previously been reported by some studies examining the combined effect of both factors on selective attention (e.g., Ahmed and De Fockert, 2012; Experiment 2).

To analyze participants’ performance in the Stroop task, mean correct RTs and error rates were again entered into two 2 × 2 ANOVAs treating congruency (congruent and incongruent) and WM load (low and high) as within-participants factors.

The ANOVA on error rates only revealed a significant main effect of prime-target congruency [*F*(1,39) = 6.15, *p* = 0.018, η^2^ = 0.14], with a reduced error rate on incongruent (*M* = 2.14) than on congruent (*M* = 3.07) trials (i.e., a reversed, strategic-Stroop effect). The RT ANOVA showed a significant congruency by WM load interaction [*F*(1,39) = 28.5, *p <* 0.001, η^2^ = 0.42], which revealed opposite behavioral effects under low and high load in the WM task. As shown in **Table 2**, when participants were required to remember series of dots forming a symmetrical pattern (low load), they could use the prime information in a strategic manner in the Stroop task, as their responses on incongruent trials were faster (by 21 ms) than on congruent trials [*t*(39) = 2.53, *p* = 0.016, *d* = 0.38]. Yet, when participants had to remember the spatial locations of dots randomly scattered on a matrix (high load), they responded slower (by 27 ms; **Table 2**) on incongruent than on congruent trials [i.e., standard interference effect; *t*(39) = 2.61, *p* = 0.013, *d* = 0.41]. This finding replicates that obtained in our Experiment 1 using a different spatial WM task, as well as the results reported by Ortells et al. (2017) with a verbal WM task.

**Table 2:** Mean (SD) correct reaction times (ms) and error percentages (in %) for congruent and incongruent trials in the Stroop task, under low and high WM load in Experiment 2.

	Prime-target congruency		
	Congruent	Incongruent	Stroop-priming
Working memory load			
Low load	530 (120.4) 3.2 (4.7)	509 (116.1) 1.9 (2.4)	+21
High load	516 (114.6) 2.7 (3.9)	543 (122.7) 2.5 (3.5)	−27

With regard to the combined effect of WM load and WMC on the strategic Stroop effect, even though the pattern of Stroop effects as a function of WM load and WMC was similar to Experiment 1, with strategic Stroop effects only being apparent in high WMC individuals who were experiencing low WM load, the three-way interaction between WM load, Congruency, and WMC did not reach significance this time (*F* < 1).

## General Discussion

In this study, we used a sequential Stroop-priming task with a differential proportion of incongruent (80%) and congruent trials (20%), which was interleaved with different types of non-verbal WM tasks demanding either a low or a high load. There were two relevant findings in our study.

Firstly, in both Experiments 1 and 2 we found a reliable WM load by congruency interaction, which revealed that participants’ performance in the Stroop-priming task was clearly influenced by WM load. When the WM task demanded a high load, participants appeared unable to strategically use the information provided by the prime word to anticipate their responses to the color target, as their responses were slower to incongruent than to congruent targets (i.e., a standard Stroop interference effect). The same Stroop interference pattern was observed across two experiments, and irrespective of whether the non-verbal WM task required participants to remember either the orientations of arrow-sets (Experiment 1) or the spatial locations of different dots displayed in a square grid (Experiment 2).

A similar Stroop congruency by WM load interaction was also reported by Ortells et al. (2017). Yet, that study manipulated WM load by means of a verbal task (i.e., memorizing sequences of digits), and one therefore cannot rule out the possibility that the absence of the strategic effect (reversed Stroop) found by these authors under a high WM load, could at least partly be attributed to verbal interference processes from the concurrent WM task. But this does not appear to be the case in the current research, especially in Experiment 2. Regarding the WM loading task used in our Experiment 1, we cannot completely rule out the possibility that participants might have employed some kind of verbal coding strategy to memorize the directions of series of arrow sets that always appeared in fixed spatial locations and ordered from left to right, similarly to verbal information. But the same argument could not be applied to the high load condition of the WM task used in Experiment 2, which required participants to memorize the spatial locations of four dots that were randomly displayed on a 16-square grid. Strategies involving verbal coding would have been unavailable for that task. Overall, the findings of Experiments 1 and 2 thus replicate and extend those reported by Ortells et al. (2017) and provide stronger tests that the effects of WM load on expectancy-based strategic process are mainly domain-general (attention control resources) rather than domain-specific (verbal interference).

On the other hand, whereas a few previous studies had examined the combined influence on performance of limiting WM resources by both loading WM and individual differences in WMC (e.g., Rosen and Engle, 1997; Kane and Engle, 2003; Ahmed and De Fockert, 2012), the interactive impact of these two factors on strategic processing of task-relevant information in selective attention had not been previously investigated.

A second key finding of our study was that the influence of loading WM on expectancy-based strategic processes was at least partially modulated by individual differences in WMC. In Experiment 1, and to some extent also in Experiment 2, we found that imposing a high load in a concurrent non-verbal WM task disrupted the implementation of expectancy-based strategies in a similar way irrespective of whether participants had an either high or low WMC (as revealed by their performance in the change localization task). Thus, when the spatial WM task demanded a high load, observers were unable to strategically use the trial probability information, and they responded slower to the incongruent than to the congruent trials (i.e., a standard Stroop interference effect) irrespective of their WMC. In clear contrast, when the WM task demanded a low load, the probability to efficiently process the task-relevant information in a strategic manner appeared to depend on WMC, as only high-WMC participants showed reliably faster responses to incongruent than to congruent targets in the Stroop-priming task. But a different result pattern was observed in low WMC individuals, who showed an opposite Stroop interference effect in Experiment 1 (and a similar pattern of effects in Experiment 2, though this time the omnibus three-way interaction was absent), even when performing the Stroop-priming task under a low WM load (**Figure 4**).

It should be noted that the reliable three-way interaction between WM load, congruency and WMC observed in Experiment 1, did not reach statistical significance in Experiment 2. Whereas the reasons for that discrepancy remain unclear, several observations seem pertinent here. First, as in Experiment 1, we also found in Experiment 2 a reliable correlation between participants’ WMC (*k* scores) and the reversed Stroop-priming effect under low WM load (*r* = 0.35, *p* = 0.028), but not under a high load. Thus, only participants with a higher WMC were able to show a reliable reversed Stroop under low load, thus replicating the findings of Experiment 1. Secondly, it is interesting to note that the overall mean WMC score for participants in Experiment 2 was higher (*k* = 3.28) than the mean score found in Experiment 1 (*k* = 3.09), with this difference being marginally significant [*t*(82) = 1.85, *p* = 0.068, *d* = 0.40]. In fact, more than half of participants in Experiment 2 included in the medium-WMC group (eight from 14 participants), could have been classified as individuals with a higher-WMC in Experiment 1. Further research addressing the combined influence of loading WM and individual differences in WMC could use an extreme-group approach. This would allow to address whether participants with WMC scores falling within the upper and lower quartiles really show a differential impact of WM load on expectancy-based strategic processes.

In order to explain the deficits in cognitive control usually shown by older adults and several clinical populations (e.g., schizophrenia patients), Braver et al. (2001, 2007) have developed the dual-mechanisms control (DMC) model (see Braver, 2012, for a review). This theory assumes that intentional or goal directed behavior can be the result of two different modes of cognitive control: proactive and reactive control. Proactive control reflects a preparatory and resource demanding type of control in which a predictive cue is used by individuals to prepare a specific response to a future target. This control mode requires active maintenance of the goal-relevant information in an accessible state, in order to efficiently focus attention on that information while ignoring competing distractors. In contrast, reactive control involves a backward-acting and less effortful process, in which the target onset would automatically induce the retrieval of the relevant information (e.g., appropriate actions) from long-term memory.

By using different tasks and experimental procedures (e.g., the AX-Continuous Performance Test, AX-CPT) to assess the DMC theory, numerous studies have reported evidence that older adults as well as younger adults with a low WMC are less likely to efficiently use a proactive cognitive control mode than young adults high in WMC (e.g., Braver et al., 2007; Hutchison et al., 2014; Redick, 2014; Richmond et al., 2015; Wiemers and Redick, 2018).

The current results fit fairly well with the DMC framework by Braver et al. (2007). Performing the Stroop-priming task with a concurrent WM task that imposed a high load could impede participants to efficiently represent the task instructions in their WM, thus explaining the absence of a strategic effect (reversed Stroop) that was observed under that WM load condition. In a similar vein, the fact that only higher WMC individuals were able to show an expectancy-based strategic effect (i.e., reversed Stroop) under a low WM load, would also be consistent with the idea that an adequate implementation of proactive control would require a high WMC, whereas participants with a low WMC are more likely to use a reactive control mode.

The observed differences between high and low WMC participants in our study also resemble those previously observed by Froufe et al. (2009) between young adults and elderly people using a similar Stroop-priming task. These authors found that only the young group were able to efficiently implement expectancy-based strategic actions under single-task conditions, and showed a reliable reversed Stroop effect. However, the older participants showed either a non-significant reversed Stroop effect, or an opposite standard Stroop interference, as occurred in the elderly group with AD. As argued by the executive attention model of WM proposed by Engle and Kane (2004) and Kane et al. (2007), having a low WMC could have a similar effect to using a WM task demanding a high load, as individuals with more limited WM resources should also show a reduced capacity for attentional control.

## Conclusion

The results of the present study, along with those recently reported by Ortells et al. (2017), clearly demonstrate that imposing a high WM load disrupts the implementation of expectancy-based strategic processes, irrespective of the nature of the concurrent WM task. Overall, these results replicate and extend recent demonstrations that reducing the availability of WM resources with a high WM load not only interferes with the ability to inhibit or suppress distracting information, but it also leads to less efficient strategic processing of task-relevant information in selective attention tasks (e.g., Heyman et al., 2014; Hutchison et al., 2014; Ortells et al., 2017; see also Kalanthroff et al., 2015).

Our study also demonstrates for first time that the effect of loading WM on expectancy-based strategies can be modulated to some extent by individual differences in WMC. Thus, an efficient implementation of facilitatory attention strategies under dual-task conditions might require that cognitive control resources are maximally available, that is, under low WM load conditions, and in high WMC individuals.

## Author Contributions

JO and JDF developed the concept and the design of the experimental work. NR, SF, and JO actively participated in the implementation of the experimental tasks, data collection, and data analyses in the two experiments. All the authors supervised the processes of accomplishing the study, contributed to writing and reviewing the manuscript, as well as to approving the final version of the manuscript.

## Conflict of Interest Statement

The authors declare that the research was conducted in the absence of any commercial or financial relationships that could be construed as a potential conflict of interest.

## Footnotes

1 A fairly similar result pattern was found in a further analysis on the Stroop-priming data, in which we included those trials with incorrect responses to the arrow memory probe. Thus, the effects of WM load [*F*(1,43) = 5.01, *p* = 0.030, η^2^ = 0.104] and congruency [*F*(1,43) = 4.49, *p* = 0.040, η^2^ = 0.095] were again significant, as well as the WM load × congruency interaction [*F*(1,43) = 6.85, *p* = 0.012, η^2^ = 0.14], and more relevant, the three-way interaction between WM load, congruency and WMC [*F*(1,42) = 5.92, *p* = 0.019, η^2^ = 0.124]. Further analyses of the latter interaction showed a crossover congruency × WMC interaction under low load [*F*(1,42) = 10.73, *p* = 0.002, η^2^ = 0.204], which showed opposite Stroop-priming effects as a function of participants’ WMC. Yet, no reliable congruency × WM interaction was found under high WM load (*F* *<* 1), such that an interference Stroop effect was always found irrespective of WMC.

2 Whereas the ANCOVA analysis considers the full range of WMC scores, for a better visual understanding of that analysis, **Figure 4** shows participants divided into high- (*k* > 3.36), medium- (*k* < 3.32), and low-WMC (*k* < 3.08) groups by using a tertile split (see Richmond et al., 2015 for a similar approach).

3 We would argue that it is highly unlikely that such a kind of verbal rehearsal could be a useful retention strategy in our experiment. Note that all of our participants were Spanish native speakers. Whereas the direction words “up,” “down,” “left,” and “right” are pronounced as monosyllabic words in English language, this is not the case regarding Spanish language, as all of those words involve three syllables (up = a-rri-ba; down = a-ba-jo; left = iz-quier-da; right = de-re-cha), Consequently, a Spanish native speaker would need much more time than an English speaker to retain in WM four direction words by using verbal rehearsal. We nonetheless decided to run Experiment 2 with a WM task that is even less likely to involve verbal coding.
